# Occurrence and abundance of ammonia‐oxidizing archaea and bacteria from the surface to below the water table, in deep soil, and their contributions to nitrification

**DOI:** 10.1002/mbo3.488

**Published:** 2017-05-19

**Authors:** Lei Zheng, Xue Zhao, Guibing Zhu, Wei Yang, Chao Xia, Tao Xu

**Affiliations:** ^1^ College of Water Sciences Beijing Normal University Beijing China; ^2^ Zhengzhou University Multi‐functional Design And Research Academy Limited Company Zhengzhou China; ^3^ State Key Laboratory of Environmental Aquatic Quality Research Center for Eco‐Environmental Sciences Chinese Academy of Sciences Beijing China; ^4^ School of Environment Beijing Normal University Beijing China; ^5^ Geological Exploration Technology Institute of Jiangsu Province Jiangsu China

**Keywords:** abundance, ammonia‐oxidizing microorganism, deep soil, nitrification rate, nitrifier, occurrence

## Abstract

Using molecular biology methods (qualitative and quantitative PCR), we determined the occurrence and abundance of ammonia‐oxidizing archaea (AOA) and ammonia‐oxidizing bacteria (AOB) from a dry inland soil in Basel, Switzerland, and from the riparian zone of Baiyangdian Lake, China. We also determined the contributions of these microorganisms to ammonia oxidization at different depths based on the nitrification rate. The number of archaeal *amo*A genes (the key functional gene in AOA) was larger than the number of bacterial *amo*A genes in each sample, suggesting a dominant role for the AOA *amo*A gene in environments with a low ammonium concentration. In Baiyangdian Lake, the number of archaeal *amo*A genes was highest at 6 m and lowest at 12 m from the land–water interface in the soil (at depths from 40 to 60 cm), close to the groundwater, which suggests that AOA become more competitive in environments with a low dissolved oxygen content and are promoted by low pH. The nitrification rate was significantly negatively correlated with depth in the Baiyangdian Lake soil and significantly positively correlated with the number of AOB *amo*A genes at this site, 6 m from the water.

## 1 INTRODUCTION

As researchers continue to study the nitrogen cycle, new reaction mechanisms and the microorganisms responsible for them are being discovered, revolutionizing our understanding of the nitrogen cycle. It was long believed that microbial oxidation of ammonia (and its hydrated ion, ammonium) was performed solely by bacteria and that only bacteria possessed the *amoA* gene that encodes ammonia monooxygenase, the key enzyme in nitrification (Jia & Conrad, 2009). However, the discovery of *amo*A in the Crenarchaeota (Venter, 2004; Wang et al., 2011) revealed the existence of an autotrophic ammonia‐oxidizing marine archaeon, *Nitrosopumilus maritimus* Könneke et al. (2005).

Microbial ammonia oxidation is the first and rate‐limiting step in nitrification, and is therefore a central process in the global nitrogen cycle that sustains life on Earth (Jia & Conrad, 2009; Wuchter et al., 2006; Falkowski, Fenchel, & Delong, 2008). Addition of the nitrification inhibitor dicyandiamide completely inhibited the nitrification activity of ammonia‐oxidizing microorganisms (Zhang 2011). The occurrence and relative abundance of ammonia‐oxidizing archaea (AOA) and ammonia‐oxidizing bacteria (AOB) vary widely in the many environments where they are found, including marine waters (Francis et al., 2005), soils(Leininger et al., 2006), bioreactors (Park et al., 2006), and hot springs(Zhang et al., 2008). There is a growing consensus that AOA may be important actors in the N cycle under unfavorable environmental conditions, such as limited nutrient availability, extreme pH, or a low dissolved oxygen content. However, it is unclear whether soil nitrification is exclusively or predominantly linked to the abundance of AOA and AOB.

Further exploration of the behavior of AOB and AOA, particularly deep in the soil, where conditions may limit the survival of other organisms, is of great importance for improving our understanding the of the soil nitrogen cycle. Hence, the aim of this study was to investigate the roles of AOA and AOB in the soil and assess the relationship between their abundance and environmental factors that change with increasing depth in the soil under conditions of high spatial heterogeneity. In this study, we compared these microorganisms in the soil of a site near Basel, Switzerland (to a depth of 1,080 cm) and a site in the riparian zone of Baiyangdian Lake, China, at distances of 6 and 12 m from the land–water interface (to a depth of 100 cm). We used techniques from molecular biology to detect the occurrence of and compare the abundance of AOA and AOB between the two sites. At the same time, we determined the contributions of these microorganisms at different depths to ammonia oxidization and the resulting effect on the nitrification rate.

## 2 EXPERIMENTAL PROCEDURES

### 2.1 Soil samples and background

We obtained three soil columns to depths below the water table at our study sites. The samples from Baiyangdian Lake were obtained at distances of 6 and 12 m from the land–water interface (hereafter, BYD‐6 and BYD‐12). The Basel sample was obtained to a depth of 1,080 cm, versus a depth of 100 cm in the two BYD samples. The depths differed between the sites because we focused on the soil from the surface to the top of the water table and then to a comparable depth below the water table. The Basel site is in north‐western Switzerland (47°33′N, 7°35′E), near the Rhine River. The Baiyangdian Lake sites (38°43′N to 39°02′N, 115°38′E to 116°07′E) were located near the largest freshwater body in northern China.

At both sites, we removed the surface litter and humus layers, then obtained samples from an area of bare soil between plants. The BYD samples were obtained at 20‐cm intervals to a depth of 100 cm. The Basel samples were obtained at 120‐cm intervals to a depth of 1,080 cm. The water table at the Basel site was about 400 cm below the surface, and that at Baiyangdian was about 50 cm below the surface. All soil samples were immediately sealed in sterile plastic bags and stored in an ice cooler until they could be returned to the laboratory. One part of each sample was stored at 4°C for soil incubation experiments, and the other was passed through a 2.0‐mm sieve and then stored at 4°C for chemical analysis, with subsamples stored at −80°C for DNA extraction to identify the soil organisms.

### 2.2 Chemical and physical properties of the soils

We extracted ammonium (NH_4_^+^‐N), nitrite (NO_2_^−^‐N), and nitrate (NO_3_^−^‐N) from the soil samples with 2mol/L KCl solution and measured their concentrations using a SAN plus continuous flow analyzer (Skalar Analytical, Breda, the Netherlands). Soil pH was determined after mixing with water at a soil to water ratio of 1:5 m/m, and we measured the loss on ignition at 550°C (LOI_550_) as a proxy for the total soil organic matter content. The other physicochemical characteristics were the soil total nitrogen (TN), total carbon (TC), and moisture content, which were measured according to standard methods (Sd, 2000). All analyses were performed on triplicate samples.

### 2.3 DNA extraction

Soil DNA was extracted from 0.33 g of freeze‐dried soil using the FastDNA Spin Kit for Soil (Qbiogene, Carlsbad, CA, USA) according to the manufacturer's protocol, with minor modifications: the contents of the Lysing Matrix E tubes were homogenized in the Bio 101 FastPrep Instrument (Bio 101, Thermo Fisher Scientific, Waltham, MA, USA) and vortexed at a speed setting of 5.5 for 45 s, and then the tubes were centrifuged at 14,000*g* for another 15 min to create pellets. Thereafter, we strictly followed the manufacturer's protocol. The DNA sediment was eluted with 75 μl of DNase/pyrogen‐free water and stored at −20°C until use. The concentrations of the extracted DNA were determined by spectrophotometric analysis using a Nano Drop 2,000 UV‐Vis Spectrophotometer (Thermo Fisher Scientific), and the quality was checked by electrophoresis on a 1% (w/v) agarose gel.

### 2.4 Polymerase chain reaction (PCR), cloning, and sequence analysis

The analysis of biodiversity can be determined using high throughput sequencing technique, like pyrosequencing and Illumina sequencing. However, it is better to use pyrosequencing than Illumina sequencing for the *amo*A gene has a longer gene fragment. But in this study, we pay attention to the occurrence and abundance not diversity of ammonia‐oxidizing archaea and bacteria from the surface to below the watertable, in deep soil, and their contributions to nitrificationin,so we use the traditional sequencing method to study the occurrenceand abundance.

PCR amplification was performed in 25‐μl reaction mixtures that included 2 ×  Go Taq Green Master Mix (Promega, Madison, WI, USA), 0.5 μl of 20 mg·ml^−1^ bovine serum albumin (Takara Bio Company, Dalian, China), 0.5 μl of each primer (10 μmol/L), and 2 μl of the DNA template diluted to 10% of its original volume (1–10 ng). We used the primer sets *amo*A1F (GGGGTTTCTACTGGTGGT)/*amo*A2R (CCCCTCKGSAAAGCCTTCTTC) (Rotthauwe, Witzel, & Liesack, 1997) and Arch‐*amo*AF (STAATGGTCTGGCTTAGACG)/Arch‐*amo*AR (GCGGCCATCCATCTGTATGT) (Francis et al., 2005) to amplify the AOB and AOA *amo*A genes, respectively. The PCR procedure followed the method of Francis et al. (2005), with minor modifications. The initial denaturation was at 95°C for 300 s, followed by denaturation at 94°C for 50 s and 39 cycles consisting of denaturation at 94°C for 110 s, annealing at 53°C for 60 s, and extension at 72°C for 60 s. The final elongation step was at 72°C for 600 s.

The PCR product was purified on a 2% w/v agarose gel and ligated into the pGEM‐T Easy Vector (Promega). The resulting ligation products were used to transform *Escherichia coli* JM109 competent cells following the manufacturer's instructions. The PCR products were screened directly for the presence of inserts using T7 (5′‐TAATACGACTCACTATAGGG‐3′) and SP6 (5′‐ATTTAGGTGACACTATAGAA‐3′) vector primers, the positive clones were selected to extract plasmid DNA using a GeneJet Plasmid Miniprep Kit (Fermentas MBI, Vilnius, Lithuania), then the amplicons were analyzed by restriction with the *Hha*I restriction endonuclease (Takara Bio). Restriction digestion was carried out in a total volume of 20 μl that included 5U of the restriction enzymes and 4 μl of the PCR products, and the system was incubated for 2 hr at 37°C.

Digested DNA fragments were analyzed by separation of the fragments on a 2% (w/v) agarose gel and visualized with a GBOX/HR‐E‐M gel documentation system (Syngene, Cambridge, UK). Representative clones from each digestion pattern were selected for sequencing using an ABI 3730XL automated sequencer (Thermo Fisher). All the sequences were analyzed, their relatives were obtained using the NCBI BLAST tool (https://blast.ncbi.nlm.nih.gov/Blast.cgi), and the sequences were aligned using the Clustal X1.83 program (Thompson et al., 1997). The sequences with at least 98% similarity were grouped using the DOTUR software (Schloss & Handelsman, 2005) and the furthest‐neighbor approach. The biodiversity was also calculated using the DOTUR software. Phylogenetic trees were constructed by the neighbor‐joining method with the Jukes–Cantor correction in the MEGA 5 software (http://www.megasoftware.net/).

### 2.5 Real‐time quantitative PCR

We quantified the abundance of the AOA and AOB *amo*A genes using an ABI 7,300 quantitative PCR instrument (Applied Biosystems, Foster City, CA, USA) with the fluorescent dye SYBR‐Green approach. Amplification was performed in 20‐μl reaction mixtures, which included 10 μl SYBR Premix Ex Taq (Takara Bio), 0.4 μl of bovine serum albumin (25 mg·ml^−1^), 0.5 μl of each primer (10 μmol/L), and 2 μl of DNA template diluted to 10% of its original volume. The amoA1F/amoA2R and Arch‐*amo*AF/Arch‐*amo*AR primer sets (Francis et al., 2005) were used to amplify the AOB and AOA *amo*A genes, respectively. The thermocycling steps for the quantitative PCR were an initial denaturation at 95°C for 30 s, followed by 40 cycles of 10 s at 95°C, 30 s at 53°C for AOA or 55°C for AOB, and 60 s at 72°C. There was no final extension.

Positive clones of *amo*A were selected to isolate plasmid DNA using a GeneJet Plasmid Miniprep Kit (Fermentas) as the gene standard. The concentration of plasmid DNA was determined using the Nanodrops ND‐1000 spectrophotometer to calculate *amo*A gene copy numbers. Standard curves were obtained with serial dilutions of the plasmid DNAs to a final value of 10% of the original concentration. The results showed an efficiency greater than 90% and a correlation coefficient of 0.98.

### 2.6 Nitrification rate of ammonia‐oxidizing microorganisms

To determine nitrification rates, we used the nitrification inhibitor dicyandiamide to decrease the soil nitrification rate and thereby substantially reduce nitrate leaching and nitrous oxide emissions (Di & Cameron, 2003; Cameron & Di, 2002). The experiment was divided into a control and three parallel experimental groups. In summary, we oven‐dried a soil sample to determine its moisture content, then added 5.0 g of fresh soil (dry weight [DW] basis) to a 50‐ml centrifuge tube containing either 1 ml of doubly distilled water or 1 ml of dicyandiamide (at 2.1 g/L), with three replicates per soil depth. All groups were incubated in the dark at 30°C, and three replicates from each treatment were destructively sampled at 0, 2, 4, and 6 days.

Aerobic conditions were maintained by opening the tubes to refresh the air every 2 days. We then extracted nitrite with 20 ml of 2 mol/L KCl, with shaking of the soil solution for 1 hr in a shaking incubator at 150 rpm and 30°C. At the end of this period, the solution was passed through a 0.45‐μm membrane filter and the nitrite content was measured with the SAN plus continuous flow analyzer.

### 2.7 Statistical analysis

The statistical analyses were conducted using correlation analysis, with Pearson's correlation coefficient for normally distributed data and Spearman's correlation coefficient for non‐normally distributed data. We used version 16.0 of the SPSS software (www.ibm.com/analytics/us/en/technology/spss/) for this analysis, and used version 12.5 of SigmaPlot (https://systatsoftware.com/products/sigmaplot/) for graphing our data. We considered results to be statistically significant at *p *<* *.05.

## 3 RESULTS

### 3.1 Chemical properties of the soil

Table 1 summarizes the chemical properties of the sampled soils as a function of depth.

**Table 1:** Chemical and physical characteristics of the soil samples

	Depth	NH_4_^+^‐N	NO_3_^−^‐N	NO_2_^−^‐N	pH	LOI_550_	Moisture content	TN	TC
	cm	mg/kg	mg/kg	mg/kg		%	%	g/kg	g/kg
Basel	0–120	2.12	4.09	0.03	7.70	3.70	27.72	1.19	45.29
	120–240	2.28	2.13	0.05	8.43	2.28	9.49	0.56	76.02
	240–360	5.63	4.43	1.21	8.05	2.14	23.38	0.47	53.71
	360–480	2.06	2.26	0.06	8.16	2.99	25.65	0.35	76.69
	480–600	2.35	3.55	0.07	8.24	0.50	15.87	0.31	76.05
	600–720	2.48	3.52	0.10	7.90	3.12	20.76	0.37	61.94
	720–840	1.66	1.09	0.05	8.61	1.96	12.81	0.30	84.91
	840–960	4.54	2.44	0.06	8.49	0.93	14.45	0.31	85.05
	960–1080	3.49	1.69	0.07	8.17	1.49	30.30	0.44	45.12
BYD‐6	0–20	2.90	2.88	0.18	7.05	5.77	42.01	1.44	23.11
	20–40	1.69	2.27	0.10	7.37	6.35	39.35	1.08	23.20
	40–60	1.27	1.78	0.07	5.90	5.43	33.02	0.78	19.52
	60–80	1.36	0.86	0.05	7.21	4.07	34.29	0.77	20.53
	80–100	1.22	1.09	0.06	6.73	4.06	30.00	0.64	17.80
BYD‐12	0–20	2.48	2.29	0.22	6.75	6.53	39.86	1.44	22.26
	20–40	1.65	3.39	0.09	6.80	4.61	37.59	1.02	19.86
	40–60	1.77	1.63	0.11	7.38	5.59	35.07	0.85	20.64
	60–80	1.58	0.86	0.07	6.91	4.34	33.75	0.64	19.98
	80–100	1.09	0.78	0.07	6.99	3.71	28.45	0.66	17.80

LOI_550_, loss of mass on ignition at 550°C (i.e., a proxy for soil organic matter content); TC, total carbon; TN, total nitrogen.

At the Basel site, the NH_4_^+^‐N content ranged from 1.66 to 5.63 mg·kg^−1^, the NO_3_^−^‐N content ranged from 1.09 to 4.43 mg·kg^−1^, and the NO_2_^−^‐N content ranged from 0.03 to 1.21 mg·kg^−1^. The highest concentrations of all three forms of N were found in the layer from 240 to 360 cm. The soil in this field was alkaline (pH ranged from 7.70 to 8.61). Soil organic matter (as measured by the LOI_550_ value) ranged from 0.50 to 3.70%. The TN content ranged from 0.30 to 1.19 g/kg, and was highest in the first layer (to a depth of 120 cm). The TC content ranged from 45.12 to 85.05 g/kg. None of the parameters showed any clear pattern as a function of depth.

In the riparian zone of Baiyangdian Lake, the NH_4_^+^‐N content ranged from 1.09 to 2.90 mg·kg^−1^, the NO_3_^−^‐N content ranged from 0.78 to 3.39 mg·kg^−1^, and the NO_2_^−^‐N content ranged from 0.05 to 0.22 mg·kg^−1^. These ranges overlapped for the BYD‐6 and BYD‐12 samples. The NO_3_^−^‐N content was highest in the 20 to 40 cm layer of the BYD‐12 soil, but concentrations of the other forms of N were highest in the first layer, and generally decreased with increasing depth. The pH was neutral to slightly acidic (ranging from 5.90 to 7.38), and was lowest in the 40–60 cm layer in BYD‐6 but highest in this layer in BYD‐12. LOI_550_ ranged from 3.71 to 6.53%, TN ranged from 0.64 to 1.44 g/kg, and TC ranged from 17.80 to 23.20 g/kg, and these ranges overlapped between the two sites. Both TN and TC decreased with increasing depth.

The soil moisture content ranged from 9.49 to 30.30% at Basel, with no clear trend as a function of depth, whereas the soil moisture content at the BYD sites ranged from 28.45 to 42.10% and generally decreased with increasing depth.

### 3.2 Occurrence of AOA and AOB in the soil

**Figure 1:**
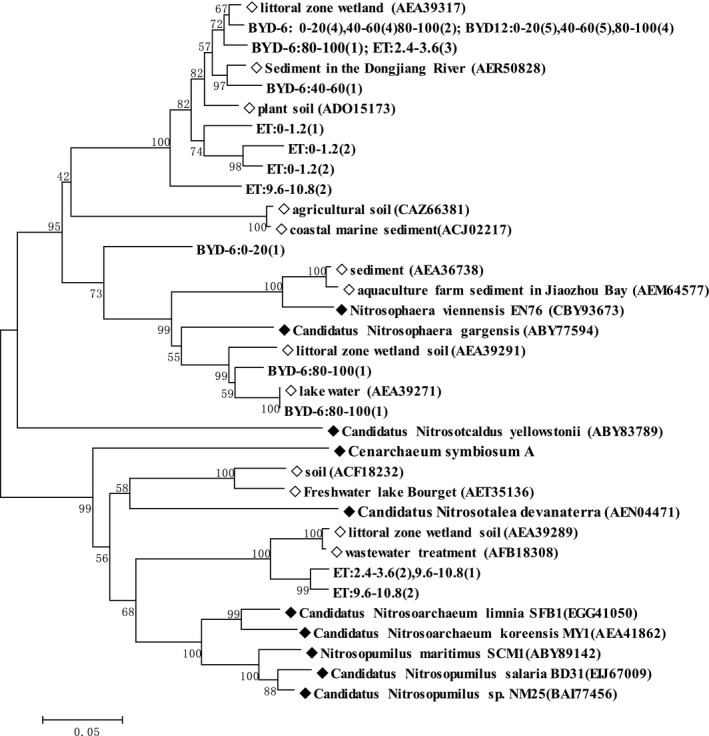
Phylogenetic tree of the amino‐acid sequences s of ammonia‐oxidizing archaea from an inland sampling site in Basel, Switzerland, and the riparian zone of Baiyangdian Lake (at 6 and 12 m from the water; respectively, BYD‐6 and BYD‐12). The numbers beside the nodes represent the bootstrap values of 1,000 replications; the scale bar at the bottom left of the phylogenetic tree represents 5 nucleotide substitutions per 100 nucleotides

**Figure 2:**
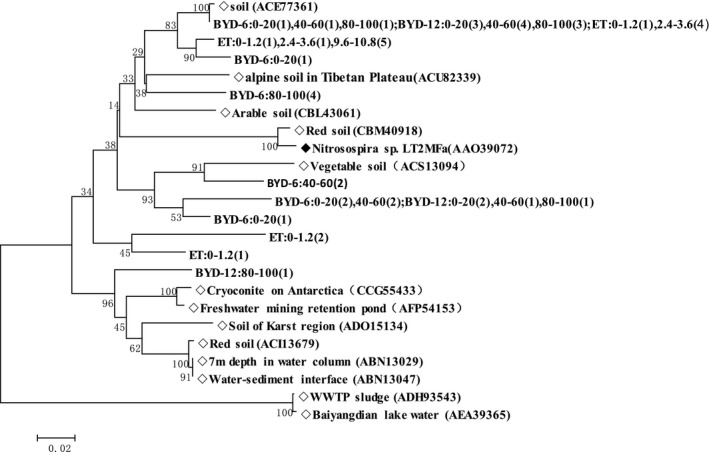
Phylogenetic tree of the amino‐acid sequences of ammonia‐oxidizing bacteria from the inland site in Basel, Switzerland, and a riparian zone site beside Baiyangdian Lake, China (at distances of 6 and 12 m from the water; respectively, BYD‐6 and BYD‐12). The numbers beside the nodes represent the bootstrap values of 1,000 replications; the scale bar at the bottom left of the phylogenetic tree represents 2 nucleotide substitutions per 100 nucleotides

To investigate the occurrence of AOA and AOB in the two soils, we tested for the bacterial and archaeal *amo*A genes. We selected three samples at each site from the surface layer (at Basel, from 0 to 120 cm; at BYD‐6 and BYD‐12, from 0 to 20 cm), three at the water table (at Basel, from 360 to 480 cm; at BYD‐6 and BYD‐12, from 40 to 60 cm), and three at the bottom of the soil column (at Basel, from 960 to 1080 cm; at BYD‐6 and BYD‐12, from 80 to 100 cm). We obtained a total of 45 archaeal *amo*A gene and bacterial *amo*A gene sequences, then used the BLAST tool to identify similar sequences; this analysis confirmed that all of the sequenced clones represented *amo*A‐like sequences. We constructed phylogenetic trees for the AOA and AOB sequences (Figures 1 and 2, respectively).

### 3.3 Abundance of AOA and AOB and nitrification rates in the soil

**Figure 3:**
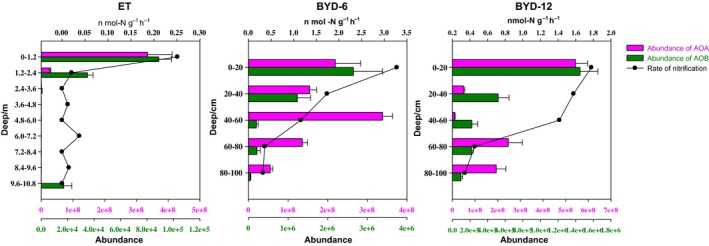
Abundance of ammonia‐oxidizing archaea and bacteria (AOA and AOB, respectively) and nitrification rates as a function of depth in the soil samples. Note that the AOB scale is two to three orders of magnitude smaller than the AOA scale. The study sites were Basel, Switzerland, and Baiyangdian Lake, China (BYD‐6 and BYD‐12 at 6 and 12 m from the water, respectively)

Figure 3 shows the abundance of AOA and AOB based on the PCR results for their *amo*A genes. At all three sites, the abundance of archaeal *amo*A was always two or three orders of magnitude higher than that of the bacterial *amo*A gene. At the two BYD sites, the archaeal *amo*A decreased initially (to a depth of 40 cm at BYD‐6 and 60 cm at BYD‐12), increased again, and then decreased to the bottom of the soil column. The highest AOA and AOB abundance at the Basel site were observed in the layer from the surface to a depth of 120 cm (3.35 × 10^8^ copies·g^−1^ DW and 8.89 × 10^4^ copies·g^−1^ DW, respectively) and decreased with depth. At BYD‐6, the abundance of AOA was highest near the water table (40–60 cm, 3.38 × 10^8^ copies·g^−1^ DW) and lowest in the bottom layer (80–100 cm, 0.55 × 10^8^ copies·g^−1^ DW). However, the abundance of AOB was highest in the top layer (0–20 cm, 2.65 × 10^6^ copies·g^−1^ DW) and decreased with increasing depth. At BYD‐12, the abundance of AOA was highest in the first layer (0–20 cm, 5.43 × 10^8^ copies·g^−1^ DW) and lowest at the water level (40–60 cm, 1.08 × 10^7^ copies·g^−1^ DW), in contrast with the results for BYD‐6. The AOB abundance was also highest in the top 20 cm of the soil (2.65 × 10^6^ copies g^−1^ DW) and decreased with increasing depth.

The nitrification rates at the Basel, BYD‐6, and BYD‐12 sites were determined during 8 day of incubations (Figure 3). All three locations had the highest nitrification rate in the surface soil, at rates of 0.251 nmol‐N g^−1^ hr^−1^ at Basel, 3.274 nmol‐N g^−1^ hr^−1^ at BYD‐6, and 1.7782 nmol‐N g^−1^ hr^−1^ at BYD‐12. The nitrification rate then decreased rapidly with increasing depth, showing little change or a slow decrease starting at a depth of 240 cm at Basel and with a steady decrease at the two BYD sites.

### 3.4 Correlation analysis for the relationships between nitrification rate and environmental variables

Because the nitrification rates and the AOA and AOB abundances at the Basel site followed non‐normal distributions, we calculated Spearman's correlation coefficient after log‐transforming the data. At the BYD‐6 and BYD‐12 sites, the data followed normal distributions, so we used Pearson's correlation coefficient, calculated with log‐transformed data (Table 2). At the Basel site, the nitrification rate was only significantly positively correlated with LOI_550_ (*r *=* *.772, *p *=* *.028). At BYD‐6, the nitrification rate was significantly positively correlated with the abundance of AOB, the contents of the three forms of nitrogen, TN, and the moisture content (*r *≥* *.9, *p *<* *.05) and was significantly negatively correlated with depth (*r *=* *−.946, *p *<* *.05). At BYD‐12, the nitrification rate was significantly positively correlated with the moisture content (*r *=* *.896, *p *=* *.040) and negatively correlated with depth (*r *=* *−.948, *p *<* *.05).

**Table 2:** Correlation analysis for the relationships between the nitrification rate and the measured environmental variables in the soil samples

	Parameter	Abundance of AOA	Abundance of AOB	Depth	NH_4_^+^ content	NO_3_^−^ content	NO_2_^−^ content	pH	LOI_550_	Moisture content	TN	TC
Basel	*r*	.409^ns^	.313^ns^	−.453 ^ns^	−.157^ns^	.235^ns^	−.376^ns^	−.444^ns^	.722*	−.009^ns^	.522^ns^	−.104^ns^
	*p*	.274	.412	.221	.412	.543	.319	.231	.028	.982	.149	.789
BYD‐6	*r*	.402^ns^	.967**	−.946*	.943*	.969**	.984**	.188^ns^	.756^ns^	.900*	.974**	.788^ns^
	*p*	.502	.007	.015	.016	.006	.003	.762	.139	.038	.005	.113
BYD‐12	*r*	.134^ns^	.716^ns^	−.948*	.796^ns^	.834^ns^	.723^ns^	−.116^ns^	.826^ns^	.896*	.863^ns^	.780^ns^
	*p*	.83	.174	.014	.107	.079	.168	.853	.085	.04	.059	.12

We used Spearman's *r* for the Basel site and Pearson's *r* for the two BYD sites. AOA and AOB, ammonia‐oxidizing archaea and bacteria, respectively; LOI_550_, loss of mass on ignition (i.e., a proxy for the organic matter content); TN, total nitrogen; TC, total carbon. *significant at *p *<* *.05; *^*^significant at *p *<* *.01, respectively (two‐tailed); ns, not significant.

## 4 DISCUSSION

The discovery of AOA has changed the traditional view of the nitrogen cycle, which formerly assumed that ammonia oxidation was completely driven by the AOB. However, the magnitude of the contribution of AOA to nitrification in soils was unknown. Previous studies reported that AOA was the dominant taxon among the ammonia‐oxidizing prokaryotes in soils and marine ecosystems with low ammonium concentrations (Leininger et al., 2006; Wuchter et al., 2006). However, other researchers observed that AOB outnumbered AOA in nitrogen‐rich sediments and in agricultural soils (Jia & Conrad, 2009; Wang et al., 2011; Wang et al., 2012). In this study, we found that AOA were much more abundant than AOB (by two or three orders of magnitude) and played an important role in ammonia oxidation.

In addition, even where the relative abundance of AOA and AOB was known, the factors that influenced their abundances were unknown. At our Basel site, which was much dryer than the two BYD sites, we only found a significant correlation with soil organic matter (LOI_550_). More parameters were significant at the two BYD sites, which were riparian sites that had much higher moisture levels. At a distance of 6 m from the water (BYD‐6), the nitrification rate was significantly positively correlated with all nitrogen parameters, as well as with the AOB abundance and soil moisture content, but negatively correlated with the depth in the soil. At 12 m from the water (BYD‐12), nitrification was significantly negatively correlated with depth and significantly positively correlated with the soil moisture content. It is interesting to note that the ammonia, as the common substrate, had a different influence on AOA and AOB population size.

We found significant levels of AOA and AOB at all three sample sites. However, based on *amo*A levels, AOA were much more abundant (by two to three orders of magnitude) than AOB. AOA may prefer low‐ammonium environments because they have a much higher affinity for ammonia than known AOB that have been cultured (Martens‐Habbena et al., 2009; Tourna et al., 2011).

Although the key factors that influence AOA and AOB are not well understood and are difficult to assess (Erguder et al., 2009), the pH value seems to be one of the most important factors based on the present results. The present results show that at all three sampling sites, soil pH was strongly correlated with the abundance of AOB and AOA. At both riparian BYD sites, the abundance of AOB decreased with increasing depth. However, AOA was most abundant at BYD‐6 and least abundant at BYD‐12 near the water table. This may be because the pH was highest in this soil layer (Table 1). An acidic environment will facilitate protonation of ammonia to produce ammonium, thereby reducing the biological availability of the substrate for these bacteria. From the perspective of substrate utilization, this may be more favorable for AOA, which are oligotrophic.

However, AOA were more abundant in soil layers where the dissolved oxygen content was low; that is, they were most abundant in the surface layer (which had a relatively high moisture content at all three sampling sites) and below the water table (i.e., where the soil was more likely to be depleted in oxygen), although we did not measure this parameter in this study because of the difficulty of such measurements. Thus, oxygen availability might be among the factors that most strongly control nitrification (Dong et al., 2011) and the abundance of AOA and AOB (Erguder et al., 2009). This hypothesis is supported by the fact that AOA can tolerate low oxygen levels (Francis et al., 2005; Könneke et al., 2005). These results suggest that AOA may be more important than AOB for nitrification in environments with limited oxygen availability.

## 5 CONCLUSIONS

In this study, we measured the nitrification rates and abundance of AOA and AOB throughout a soil column that extended from the soil surface to well below the water table. At all three sampling sites, AOA and AOB were most abundant near the surface of the soil column and at the bottom layer, below the water table. However, AOA were much more abundant than AOB in every soil layer, suggesting that they may play a key role in determining nitrification rates. Their abundance may be affected by multiple factors, including the dissolved oxygen content, soil solution pH, and availability of their preferred nitrogen substrates. Our results, combined with those of previous researchers, suggest that the AOA are better adapted to a soil environment with low nutrient and oxygen contents and low pH. In the riparian zone of Baiyangdian Lake, which is a much wetter environment than that at the Basel site, AOB played a more important role in the ammonia oxidation process than at the Basel site, despite being less abundant than the AOA.

## CONFLICT OF INTEREST

There are no conflicts of interest.
